# Effects of Sublethal Exposure to *Piper aduncum* L. Essential Oil on Population Growth of Stored-Product Insect Pests

**DOI:** 10.3390/insects17090951

**Published:** 2026-09-12

**Authors:** Gabriela da Silva Tamwing, Marcelo Luan Costa Machado, Adalberto Hipólito de Sousa

**Affiliations:** 1 Center of Biological and Natural Sciences, Universidade Federal do Acre, Rio Branco 69920900, AC, Brazil; gabrielat.agro@gmail.com (G.d.S.T.); marcelolcmachado@gmail.com (M.L.C.M.); 2 Department of Plant Science, Agricultural Science Center, Universidade Federal do Paiuí, Teresina 64049550, PI, Brazil

**Keywords:** alternative control, storage, botanical insecticides, reproduction

## Abstract

Botanical insecticides, such as essential oils, have drawn increasing interest in pest management. However, sublethal effects on exposed individuals and their offspring are often overlooked. This study evaluated the population growth of *Sitophilus zeamais*, *Oryzaephilus surinamensis*, *Cryptolestes ferrugineus*, and *Tribolium castaneum* in maize grains exposed to sublethal concentrations (1/3 and 2/3 of the LC_50_) of *Piper aduncum* L. essential oil (PAEO). Population growth was assessed using daily emergence curves, total adult emergence, and grain mass loss. Sublethal PAEO exposure reduced and delayed population growth of *S. zeamais*, *C. ferrugineus*, and *T. castaneum*. At 2/3 of the LC_50_, total adult emergence decreased by 42.6% in *S. zeamais* and 40.2% in *T. castaneum* compared with the control. In contrast, *O. surinamensis* showed an accelerated and increased emergence response, with a maximum of 10.67 ± 1.24 adults day^−1^ at 2/3 of the LC_50_, representing a 174.3% increase relative to the control, consistent with a possible hormetic response. These findings demonstrate species-specific responses to sublethal PAEO exposure, with population growth suppressed in three species but stimulated in *O. surinamensis*. This highlights the importance of appropriate dose selection to maximize pest suppression and avoid unintended stimulatory effects under stored-grain conditions.

## Simple Summary

Plant essential oils have increasingly been studied as alternatives to conventional insecticides for the control of pests that attack stored grains. In addition to being naturally derived, these products may reduce the environmental impacts associated with synthetic insecticides. However, even when they do not cause insect mortality, low doses of these compounds can affect insect development and reproduction. In this study, we evaluated the effects of sublethal concentrations of *Piper aduncum* essential oil on four important stored-grain pests *Sitophilus zeamais*, *Cryptolestes ferrugineus*, *Oryzaephilus surinamensis* and *Tribolium castaneum*. The results showed that the essential oil reduced and delayed population growth in three of these species, helping to decrease grain damage. However, for *O. surinamensis*, the treatment accelerated and increased adult emergence, indicating a phenomenon known as hormesis, in which low doses of a substance can stimulate an organism’s development. These findings demonstrate that *Piper aduncum* essential oil has potential for the management of stored-grain pests, but they also highlight the importance of using appropriate doses, since improper applications may favor the development of certain pest species.

## 1. Introduction

Controlling insect pests in stored grains represents a crucial challenge in agriculture, as preserving product quality is fundamental for food security and economic sustainability [1]. Grain storage is susceptible to infestation by various insect pests, including *Sitophilus zeamais* Motschulsky (Coleoptera: Curculionidae), *Oryzaephilus surinamensis* (L.) (Coleoptera: Silvanidae), *Cryptolestes ferrugineus* (Stephens) (Coleoptera: Cucujidae), and *Tribolium castaneum* (Herbst) (Coleoptera: Tenebrionidae). These species cause significant damage, resulting in irreversible quantitative and qualitative losses [2].

In storage facilities, these pests are primarily controlled through the application of synthetic insecticides, which are generally recognized as fast and effective [3,4]. However, the irrational use of pesticides has raised concern due to health problems, environmental impacts, and the development of resistant populations [5,6]. In this context, botanical insecticides, such as plant extracts and essential oils, have emerged as promising alternatives for controlling stored-grain pests [7,8,9].

Botanical insecticides not only cause mortality but may also alter insect behavior [10]. Such changes may promote adaptive responses and increase the survival rate under exposure to toxic compounds [11,12], potentially leading to the selection of resistant populations over time [4,13].

Although the lethal effects of essential oils are well documented [14], little is known about their sublethal effects on insect physiology and behavior [15]. Ref. [16] reported that adults of *S. zeamais*, *S. oryzae*, and *C. ferrugineus* exhibited reduced flight activity after exposure to sublethal concentrations of *Piper aduncum* L. essential oil (PAEO). Similarly, sublethal concentrations of *Piper hispidinervum* essential oil decreased the instantaneous growth rates and emergence of different populations of *S. zeamais* [17].

These observations indicate that exposure to sublethal concentrations of insecticides can alter important biological traits in insects, including development time, longevity, fertility, fecundity, oviposition, locomotion, and feeding behavior [18,19,20,21,22]. Understanding these effects is crucial for resistance management in pest populations [12,23].

Therefore, investigating the effects of sublethal exposure to botanical insecticides on insect reproductive performance is essential to prevent failures in stored-product pest management that may lead to the development of resistant populations or outbreaks of secondary pests [24]. The aim of this study was to evaluate the effects of sublethal exposure to PAEO on the population growth of *S. zeamais*, *O. surinamensis*, *C. ferrugineus*, and *T. castaneum*.

## 2. Materials and Methods

Insect rearing and the bioassays were conducted at the Integrated Pest Management Laboratory of the Federal University of Acre (Universidade Federal do Acre, UFAC). PAEO extraction was performed at the Natural Products Laboratory of the Technology Foundation of the State of Acre (Fundação de Tecnologia do Estado do Acre, FUNTAC).

### 2.1. Insect Rearing

Specimens of *S. zeamais*, *O. surinamensis*, *C. ferrugineus*, and *T. castaneum* were initially collected in Rio Branco, Acre, Brazil. They were reared in 1.5-L glass jars lined with organza fabric and covered with perforated plastic lids, under controlled conditions of temperature (27 ± 2 °C), relative humidity (70 ± 5%), and continuous scotophase (24 h).

Whole maize kernels (13% moisture content, wb) were used as a food substrate for the primary pest (*S. zeamais*), whereas crushed kernels were provided for the secondary pests (*C. ferrugineus*, *O. surinamensis*, and *T. castaneum*). The grains were previously fumigated with phosphine (PH_3_) and kept refrigerated to prevent re-infestation.

### 2.2. Obtaining and Extracting Piper Aduncum Essential Oil

Leaves of *P. aduncum* were collected from adult plants at the UFAC campus in the municipality of Rio Branco, Acre, Brazil (9°57′34.9″ S, 67°51′30.6″ W). A voucher specimen was deposited in the UFACPZ Herbarium of UFAC under registration number UFACPZ 20.646. The species identification was confirmed by Dr. Elsie Franklin Guimarães, member of the herbarium of the Rio de Janeiro Botanical Garden (RB Herbarium).

The plant material was gathered in August 2021 during the morning hours. Leaves were collected from branches approximately 0.4 m above ground level and then dried at 45 °C until constant mass was achieved. Afterward, the material was manually broken into smaller fragments and stored in 10-kg plastic bags until extraction.

PAEO was extracted by hydrodistillation using a heating mantle coupled to a 5-L round-bottom flask and a Clevenger-type apparatus. Each extraction used 150 g of dried leaves mixed with 2 L of distilled water. After the procedure, the oil phase was separated using a separatory funnel and dried over anhydrous sodium sulfate ≥ 99%, purchased from Synth (São Paulo, SP, Brazil). Finally, the resulting PAEO was stored in amber glass vials and maintained at 4 °C in a biochemical oxygen demand (BOD) incubator.

### 2.3. Piper Aduncum Essential Oil Composition

Gas chromatography–mass spectrometry (GC–MS) was carried out at the Department of Technical and Scientific Police of the Institute of Forensic Analysis (Instituto de Análises Forense, IAF), Civil Police of Acre State (Polícia Civil do Acre). The GC–MS system used was an Agilent Technologies model 7890A/5975C.

PAEO was diluted to 2% in methanol and injected into an HP-5MS capillary column (30 m × 0.25 mm internal diameter × 0.25 μm film thickness), using helium (He) as the carrier gas under split injection mode. The injector temperature was initially set at 290 °C. The oven temperature program started at 80 °C for 5 min, followed by an increase of 4 °C min^−1^ to 285 °C, which was maintained for 40 min. Both the detector and the system interface were maintained at 290 °C, and the mass detector operated under electron impact ionization at 70 eV. Mass spectra were recorded over a scan range of 30–600 Da. The chemical constituents were identified by comparing their mass spectra with those in the equipment library and further confirmed using the literature on essential oils [25] and the National Institute of Standards and Technology (NIST) database. For this purpose, the linear retention index (LRI) relative to the n-alkane series (C10–C40, Fluka Analytical) and the Kovats index (KI) were calculated.

### 2.4. Population Growth Bioassays

The tests were conducted separately for each species. The experimental units consisted of 1.0 L plastic flasks covered with perforated lids to allow air exchange and containing 200 g of maize kernels (13% moisture content, wb) treated with sublethal concentrations of PAEO corresponding to 1/3 and 2/3 of the LC_50_ for the respective species (Table 1). The LC_50_ values were estimated from concentration–response curves, and Probit analysis of mortality data was performed using SAS software version 9.0 (SAS Institute, Minato, Japan).

The solutions were applied to the grain mass using a dual-action airbrush with internal mixing and a gravity-feed system (model BC 60, Steula, Sao Paulo, Brazil). Spraying was performed at a working pressure of 15 psi, applying 400 μL of the solution per 200 g of maize [26].

After this procedure, the maize kernels were infested with 50 unsexed adult insects aged up to 48 h after emergence. Subsequently, the flasks were stored in BOD incubators under constant conditions of temperature (25 ± 2 °C) and relative humidity (70 ± 5%). The experiment was performed in a completely randomized design with four replications. The control treatment consisted of pure acetone.

Eleven days after infestation, all insects were removed from the flasks according to the method described by [27]. The adult offspring emerging from the feeding substrate were counted and removed on alternate days over the entire emergence period, with the last three counts recorded as zero. Daily emergence data were analyzed using only observations from alternate-day assessments, due to the influence of sampling intervals [27,28].

### 2.5. Total Number of Emerged Insects and Grain Mass Loss

The total number of emerged insects was recorded at the end of the emergence period. Additionally, grain mass loss was measured using a precision balance and calculated according to the following equation:

$$Mass\;loss\left(\%\right)=\frac{Mi-Mf}{Mi}\;\times \;100$$

where *Mi* = initial mass (g) and *Mf* = final mass (g)

### 2.6. Statistical Analyses

Daily and normalized cumulative emergence data were analyzed by non-linear regression using the curve-fitting function in SigmaPlot software version 13.1 (Systat Software, Inc., San Jose, CA, USA). The variables total number of emerged insects and grain mass loss were subjected to analysis of variance (PROC GLM; SAS Institute, version 9.0). The experiments followed a completely randomized design with four replicates. Tukey’s test (*p* < 0.05) was subsequently used to compare the species and the two sublethal concentrations of PAEO, using SISVAR software, version 6.5.

## 3. Results

### 3.1. Piper Aduncum Essential Oil Composition

The analysis by GC–MS identified compounds belonging to the phenylpropanoid and sesquiterpene classes in PAEO. Dillapiole was the major component (70.34%), followed by myristicin (9.57%), viridiflorol (4.68%), (E)-caryophyllene (3.82%), caryophyllene oxide (2.38%), and (+) spathulenol (2.36%) (Table 2).

### 3.2. Population Growth Rate

**Figure 1:**
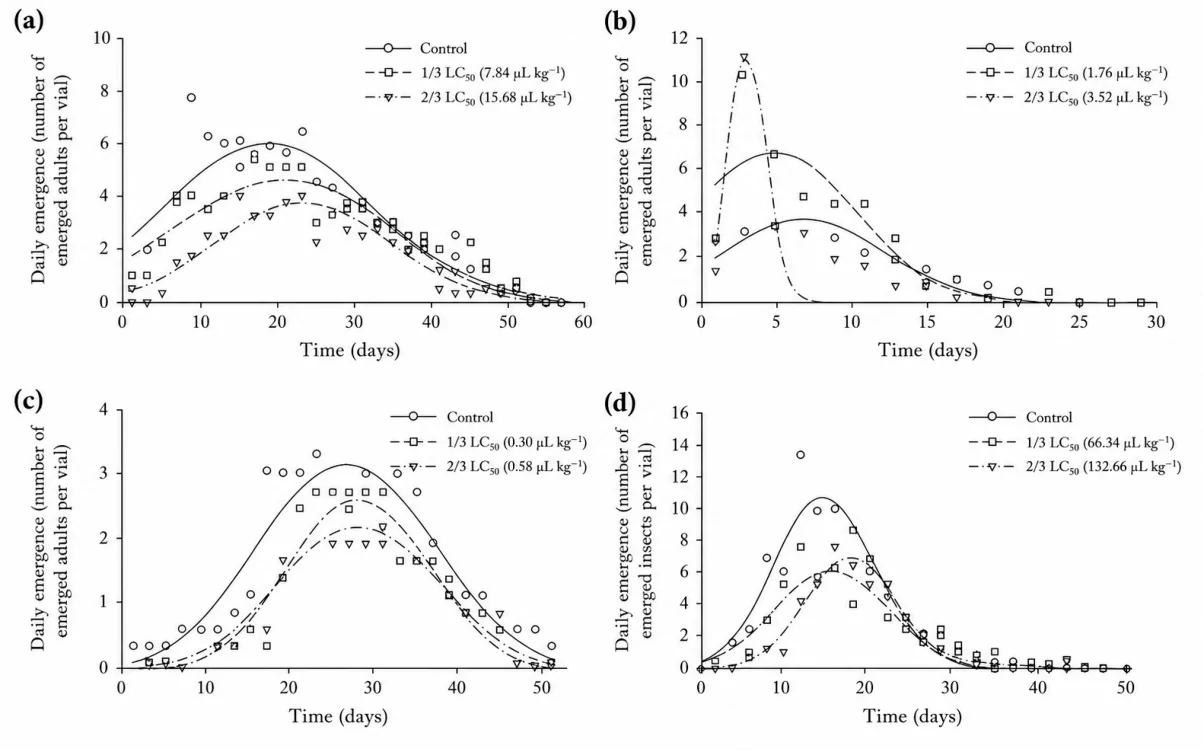
Daily emergence of adults of *Sitophilus zeamais* (**a**), *Oryzaephilus surinamensis* (**b**), *Cryptolestes ferrugineus* (**c**), and *Tribolium castaneum* (**d**), either unexposed (control) or exposed to sublethal concentrations of *Piper aduncum* essential oil. Symbols represent the mean of four replicates.

The Gaussian peak model with three parameters y (x) = a exp (−0.5 ((x − b)/c)^2^) provided the best fit to the daily emergence data for the four stored-grain pest species (*S. zeamais*, *O. surinamensis*, *C. ferrugineus*, and *T. castaneum*) (*p* < 0.0001; R^2^ > 0.91; Figure 1; Table 3). Variations in the estimated parameters were influenced by exposure to sublethal concentrations of PAEO, and the daily emergence regression curves highlighted differences in population growth rates among the species (Figure 1; Table 3).

Treatment of maize grains with sublethal concentrations of PAEO delayed the peak emergence of *S. zeamais*, *T. castaneum*, and *C. ferrugineus* adults (Figure 1a,c,d), with little overlap relative to the control. In contrast, a distinct pattern was observed for *O. surinamensis*, in which the sublethal concentration equivalent to 2/3 of the LC_50_ accelerated the peak emergence of adults. Notably, emergence declined after the curve inflection point, and offspring emergence ceased approximately seven days after the first occurrence (Figure 1b).

Lower estimates for the daily emergence peak (parameter a) were observed for *S. zeamais*, *T. castaneum*, and *C. ferrugineus* exposed to sublethal concentrations of PAEO, indicating reduced adult emergence. Contrary to expectations, *O. surinamensis* exhibited a significantly higher maximum emergence peak (10.67 ± 1.24 emerged adults day^−1^) at 2/3 of the LC_50_, representing an increase of approximately 174.29% relative to the control (Table 3).

Estimates for the number of days required to reach peak emergence (parameter b) were higher for *S. zeamais*, *T. castaneum*, and *C. ferrugineus* exposed to sublethal concentrations of PAEO, indicating delayed emergence. In contrast, *O. surinamensis* reached its population peak only three days after the first emergence when grains were treated at 2/3 of the LC_50_ (Table 3).

### 3.3. Total Number of Emerged Insects and Grain Mass Loss

**Figure 2:**
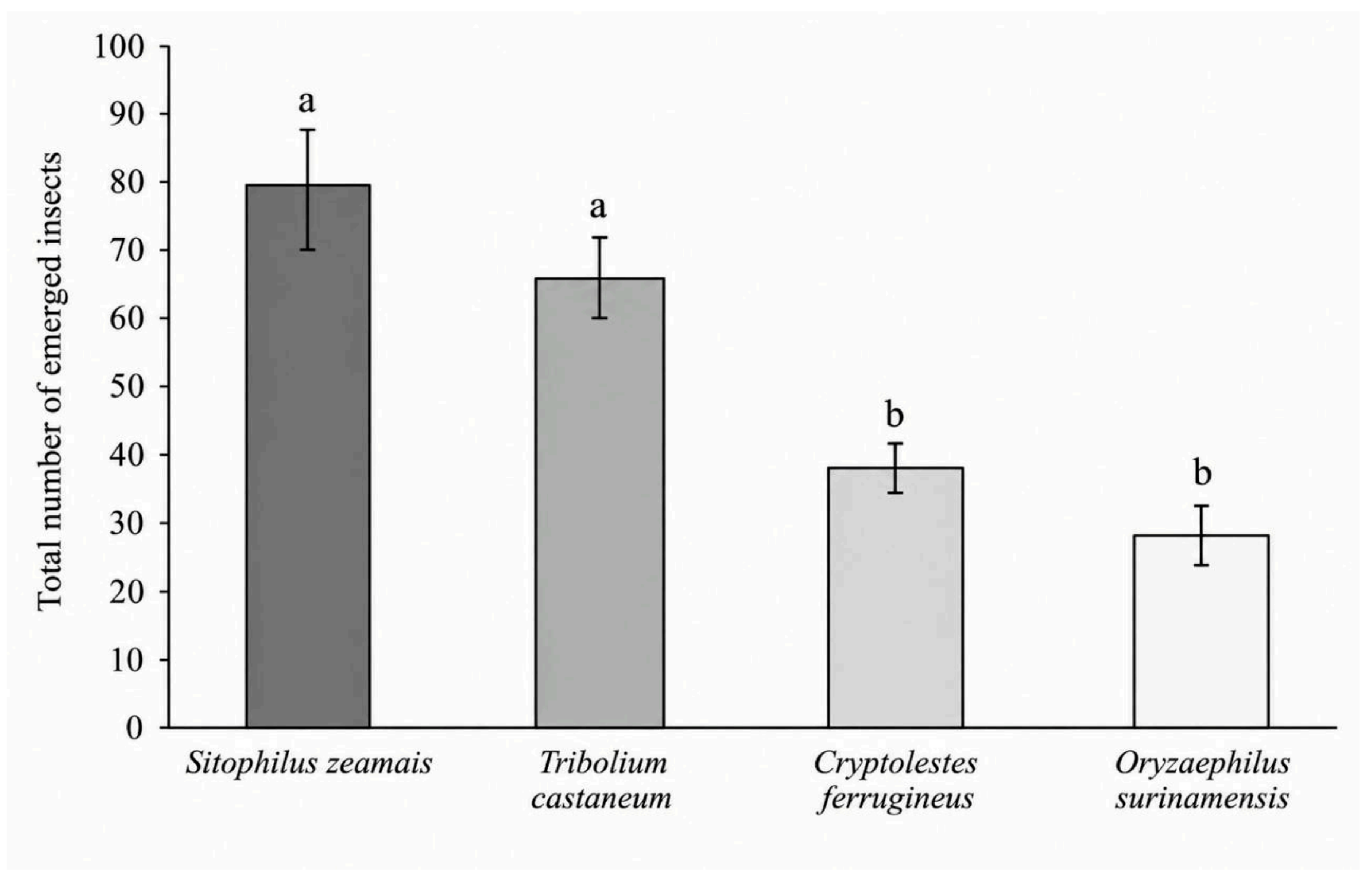
Total number of emerged adults of *Sitophilus zeamais*, *Tribolium castaneum*, *Cryptolestes ferrugineus*, and *Oryzaephilus surinamensis*. Bars followed by different letters are statistically different according to Tukey’s test (*p* < 0.05).

Significant differences in the total number of emerged insects were observed among the species and sublethal concentrations of PAEO (F3,44 = 24.51, *p* < 0.0001) (Figure 2). *S. zeamais* and *T. castaneum* showed greater adult emergence (78.75 ± 11.02 and 65.08 ± 5.16, respectively) than *C. ferrugineus* (38.17 ± 2.79) and *O. surinamensis* (28.33 ± 6.09).

A significant effect of sublethal concentrations of PAEO on the total number of emerged adults was observed for *S. zeamais* (F2,9 = 7.17, *p* < 0.01) and *T. castaneum* (F2,9 = 11.53, *p* < 0.01) (Table 4). Exposure to PAEO at 2/3 of the LC_50_ reduced the number of emerged insects by 42.6% in *S. zeamais* and 40.2% in *T. castaneum*. In contrast, no significant effect of this treatment was observed for *C. ferrugineus* (F2,9 = 3.27, *p* > 0.01) and *O. surinamensis* (F2.9 = 3.22, *p* > 0.01) (Table 4).

Regarding grain mass loss, no significant effect of sublethal concentrations of PAEO was verified for any of the species studied (*p* ≥ 0.45).

## 4. Discussion

Sublethal exposure to PAEO negatively affected the population growth rates of *S. zeamais*, *C. ferrugineus*, and *T. castaneum*. Previous studies have reported the influence of sublethal exposure to essential oils and their components on the emergence of stored-grain pest populations [13,29,30]. The sublethal effect of PAEO on *S. zeamais*, *C. ferrugineus*, and *T. castaneum* may result from reduced female oviposition [30] or from the ovicidal or larvicidal activity of the bioinsecticide, as previously reported for the essential oils of *Croton pulegiodorus* Baill (Euphorbiaceae), *Croton heliotropiifolius* Kunth (Euphorbiaceae), and *Ocimum basilicum* L. (Lamiaceae) against *T. castaneum* [31].

Insects exposed to sublethal concentrations of insecticides, whether botanical or synthetic, exhibit behavioral and physiological changes, including fewer viable eggs, shorter oviposition period, lower larval and pupal weights, reduced adult emergence, shorter longevity, and less fertility. These disturbances in the reproductive system, even under sublethal treatments, can have significant consequences for population dynamics across generations [32].

Although sublethal exposure to PAEO significantly reduced the population growth of *S. zeamais, C. ferrugineus*, and *T. castaneum*, these concentrations were not capable of completely eradicating these species. In addition, no significant reduction in grain mass loss was detected under the experimental conditions, indicating that the observed effects on population dynamics should not be directly interpreted as equivalent to effective protection of stored products. Thus, understanding the sublethal effects of insecticides is fundamental, given that insects are constantly exposed to sublethal concentrations, particularly in stored grains [11]. However, studies on the topic are still incipient, especially regarding new compounds. From a practical perspective, these findings suggest that PAEO should be considered as a potential component of integrated pest management rather than as a standalone control measure. Its practical effectiveness will depend on its ability to maintain biologically effective concentrations throughout storage and across the entire grain mass.

Exposure to insecticides can alter mortality, emergence, immigration, and emigration patterns, thereby affecting insect population dynamics. The magnitude of these effects may vary among genotypes [33]. In the present study, sublethal concentrations of PAEO enhanced the population growth of *O. surinamensis* relative to the control, as evidenced by higher daily emergence rates and a shorter time required to reach peak emergence. This result has important practical implications because differences in exposure within a stored-grain mass may lead to areas where insect populations are insufficiently controlled.

This stimulatory response may be related to hormesis, a phenomenon characterized by the beneficial effects of low concentrations of compounds that are toxic at higher levels [11,20]. Evidence of hormetic responses has also been reported in other stored-product pests. In S. zeamais, for example, ref. [34] reported that the emergence of adults was favored under sublethal concentrations of deltamethrin and spinosad. Similarly, ref. [35] found that sublethal concentrations of essential oils led to earlier emergence in this species, as well as increased grain consumption and, consequently, resulted in greater body mass. The mechanisms underlying hormetic responses induced by insecticides are still not fully understood [20,36]. However, the responses observed under sublethal exposure may result from compensatory biological processes, whereby exposed adults allocate energy resources to offspring production at the expense of self-maintenance [20,37].

Overall, such responses may compromise pest management strategies by favoring the resurgence or outbreak of secondary pests [19,38,39]. Therefore, understanding the sublethal effects of synthetic and natural insecticides should be a major focus of toxicological studies, as sublethal exposures are likely more frequent in storage environments than lethal ones, whether due to natural degradation or non-uniform application [33]. This issue is particularly relevant to botanical insecticides such as PAEO because non-uniform application may result in areas receiving concentrations below the effective level, potentially increasing the frequency of sublethal exposure. In the case of *O. surinamensis*, such exposure is of particular concern because the concentrations evaluated in the present study were associated with increased and earlier adult emergence, consistent with a possible hormetic response. Regardless of the type of insecticide used, both lethal and sublethal effects must be fully explored, and this information should be incorporated into integrated pest management decision-making [17].

Based on the results, the present study showed that PAEO, even at sublethal concentrations, delayed and reduced the population growth of *S. zeamais*, *C. ferrugineus*, and *T. castaneum*. On the other hand, sublethal exposure to PAEO may have induced hormesis in *O. surinamensis*, which may compromise the effectiveness of this bioinsecticide by increasing pest populations and potentially favoring the selection of resistance alleles [19]. Therefore, these findings reinforce the need to establish application rates that provide sufficient pest suppression while minimizing the occurrence of sublethal exposure.

Overall, before PAEO can be recommended for commercial or operational use, semi-commercial and commercial-scale studies should evaluate application uniformity, formulation stability, persistence, dose–response relationships under different temperature and relative humidity conditions, effects on grain quality and sensory characteristics, economic feasibility, and the potential occurrence of sublethal or hormetic responses. These assessments will be essential to determine whether the population-level effects observed under laboratory conditions can be translated into consistent and effective protection of stored grains under real storage conditions.

## 5. Conclusions

Sublethal exposure to PAEO reduced and delayed the population growth of *S. zeamais*, *C. ferrugineus*, and *T. castaneum*, *S. zeamais* and *T. castaneum* showed the highest overall adult emergence. In contrast, sublethal concentrations of PAEO (1.76 and 3.52 μL kg^−1^) stimulated the population development of O. *surinamensis*, which may compromise the effectiveness of the bioinsecticide and potentially favor the development of resistant populations over time. Despite these effects on population emergence dynamics, PAEO did not significantly reduce grain mass loss under the experimental conditions. Overall, the results demonstrate that PAEO produces species-specific sublethal effects and that concentrations below lethal levels may either suppress or stimulate pest population development. Therefore, appropriate dose selection and further evaluation under storage conditions are essential to ensure effective pest suppression and avoid unintended stimulatory responses, particularly in *O. surinamensis*.

## Figures and Tables

**Table 1:** Sublethal concentrations of *Piper aduncum* essential oil (PAEO) (μL kg^−1^) used in population growth bioassays.

Insect Species	Sublethal Concentrations of PAEO (μL kg^−1^)	
	1/3 LC_50_	2/3 LC_50_
*Sitophilus zeamais*	7.84	15.68
*Oryzaephilus surinamensis*	1.76	3.52
*Tribolium castaneum*	66.34	132.66
*Cryptolestes ferrugineus*	0.30	0.58

**Table 2:** Chemical composition and relative concentration of the compounds identified in *Piper aduncum* essential oil using gas chromatography–mass spectrometry analysis (GC–MS).

Components	Class	LRI ^1^	KI ^1^	LRI ^2^	KI ^2^	Relative Concentration (% Area)
α-copaene	Sesquiterpene	1383	1386	1374	1376	0.96
(E)-caryophyllene	Sesquiterpene	1429	1433	1417	1419	3.82
α-humulene	Sesquiterpene	1464	1470	1452	1454	1.04
Germacrene-D	Sesquiterpene	1492	1499	1484	1485	1.42
Pentadecane	Sesquiterpene	1504	1511	1500	1500	1.20
Myristicin	Phenylpropanoid	1534	1539	1517	1518	9.57
(+) spathulenol ^3^	Sesquiterpene	1591	1592	-	-	2.36
Caryophyllene oxide	Sesquiterpene	1595	1596	1582	1583	2.38
Viridiflorol	Sesquiterpene	1605	1606	1592	1592	4.68
Dillapiole	Phenylpropanoid	1650	1653	1620	1620	70.34
Apiol	Phenylpropanoid	1698	1703	1677	1678	1.15
Total						98.92 (identified)

^1^ Calculated according to the n-alkane series standard; ^2^ identified based on the literature [25]; ^3^ identified using the NIST database; LRI: linear retention index; KI: Kovats index.

**Table 3:** Non-linear regression analyses of population growth curves for *Sitophilus zeamais*, *Oryzaephilus surinamensis*, *Cryptolestes ferrugineus*, and *Tribolium castaneum*.

Variable	Model	Species	Treatment	Estimated Parameters (±SEM)			df_error_	*F*	*R* ^2^
				*a*	*b*	*c*			
Daily emergence	*y (x)* = *a exp* (−0.5 ((*x* − *b*)/*c*)^2^)	S. *zeamais*	Control	6.16 ± 0.33	18.59 ± 0.92	13.79 ± 1.03	26	74.69	0.92
			1/3 LC_50_	4.83 ± 0.25	20.62 ± 0.95	14.87 ± 1.07	26	68.28	0.92
			2/3 LC_50_	3.99 ± 0.21	23.30 ± 0.70	11.68 ± 0.73	26	30.08	0.94
		*O. surinamensis*	Control	3.89 ± 0.30	6.93 ± 0.52	5.32 ± 0.59	12	56.97	0.95
			1/3 LC_50_	6.86 ± 0.80	4.87 ± 1.13	5.52 ± 1.19	12	27.43	0.91
			2/3 LC_50_	10.67 ± 1.24	3.12 ± 0.22	1.31 ± 0.16	12	30.08	0.91
		*C. ferrugineus*	Control	3.16 ± 0.14	26.68 ± 0.58	11.17 ± 59	23	96.32	0.95
			1/3 LC_50_	2.66 ± 0.14	28.11 ± 0.55	8.95 ± 0.55	23	100.53	0.95
			2/3 LC_50_	2.26 ± 0.15	28.16 ± 0.76	10.27 ± 0.78	23	55.76	0.91
		*T. castaneum*	Control	10.82 ± 0.61	15.65 ± 0.40	6.05 ± 0.40	21	118.62	0.96
			1/3 LC_50_	6.44 ± 0.43	16.48 ± 0.52	6.80 ± 0.53	21	75.74	0.94
			2/3 LC_50_	7.29 ± 0.28	18.82 ± 0.24	5.33 ± 0.24	21	281.93	0.98

All estimated parameters were significant at *p* < 0.05 according to Student’s *t*-test. All models were significant at *p* < 0.05 according to the F-test.

**Table 4:** Total number of emerged insects in response to exposure to sublethal concentrations of *Piper aduncum* essential oil (PAEO).

Species	Sublethal Concentration of PAEO (μL kg^−1^)	Total Number of Emerged Insects
*S. zeamais*	Control	98.00 ± 7.87 a
	1/3 LC_50_ (7.84 μL kg^−1^)	82.00 ± 7.87 ab
	2/3 LC_50_ (15.68 μL kg^−1^)	56.25 ± 7.87 b
*T. castaneum*	Control	86.25 ± 5.47 a
	1/3 LC_50_ (66.34 μL kg^−1^)	57.50 ± 5.47 b
	2/3 LC_50_ (132.66 μL kg^−1^)	51.50 ± 5.47 b
*C. ferrugineus*	Control	42.00 ± 3.67 a
	1/3 LC_50_ (0.30 μL kg^−1^)	30.50 ± 3.67 a
	2/3 LC_50_ (0.58 μL kg^−1^)	42.00 ± 3.67 a
*O. surinamensis*	Control	23.00 ± 4.27 a
	1/3 LC_50_ (1.76 μL kg^−1^)	37.50 ± 4.27 a
	2/3 LC_50_ (3.52 μL kg^−1^)	26.00 ± 4.27 a

Means followed by the same lowercase letter in the column do not differ statistically (*p* < 0.05).

## Data Availability

The datasets used and/or analysed during the current study available from the corresponding author on reasonable request.

## Abbreviations

The following abbreviations are used in this manuscript:

PAEO	*Piper aduncum* L. essential oil
LC_50_	Lethal concentration capable of killing 50% of the insects
LC_95_	Lethal concentration capable of killing 95% of the insects
